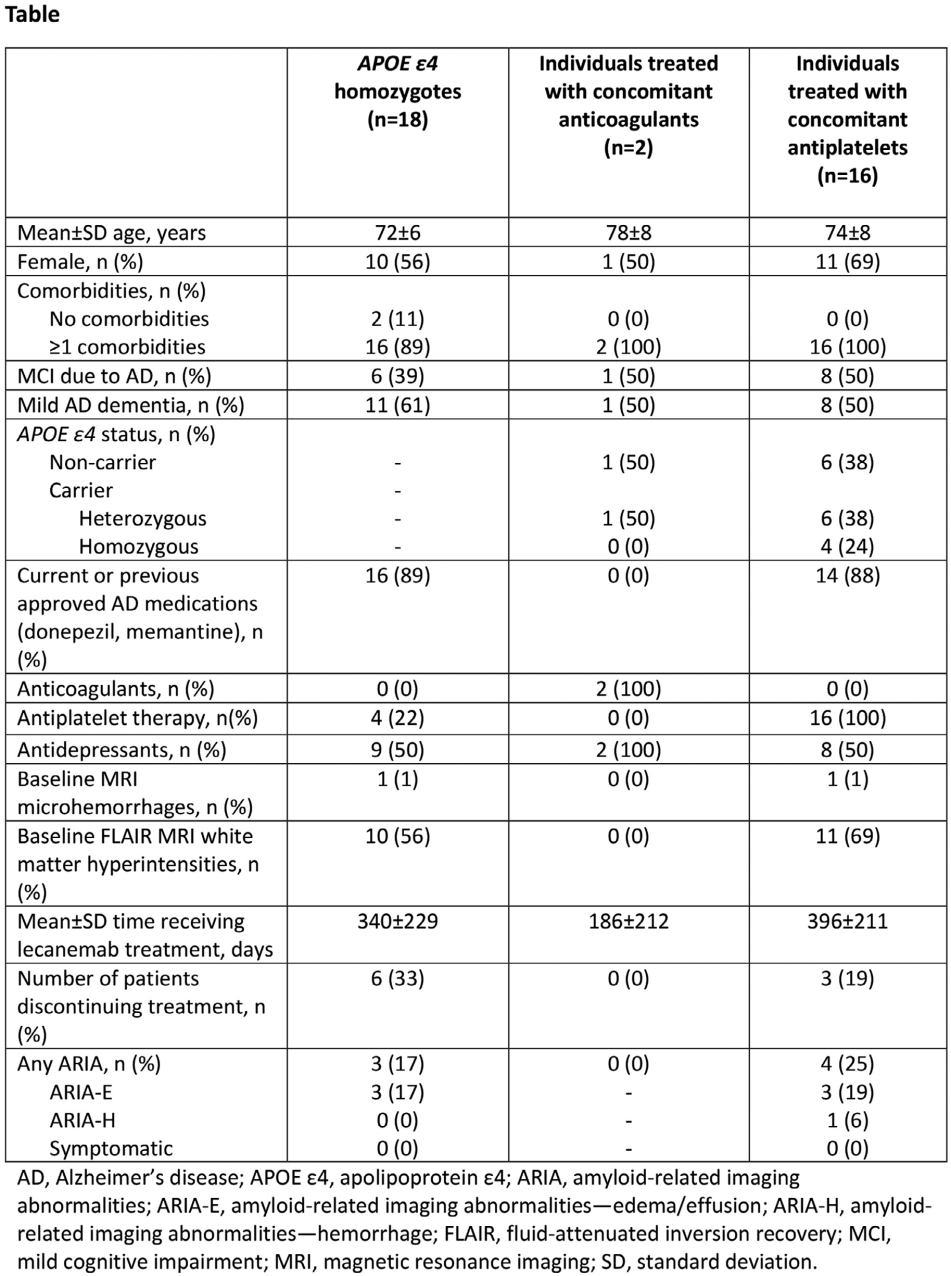# Real‐World Use of Lecanemab in APOE ε4 Homozygotes and in Patients on Antithrombotic Therapy

**DOI:** 10.1002/alz70861_108603

**Published:** 2025-12-23

**Authors:** Marwan N. Sabbagh, David C Weisman, Michael Henry Rosenbloom, Jose Soria‐Lopez, Gregory Cooper, Samuel Giles, Cara Leahy, Martin Sadowski, Curtis Schreiber, Paul E Schulz, Christian J Camargo, Brooke Allen, Courtney Adams, Daryl Jones

**Affiliations:** ^1^ Barrow Neurological Institute, Phoenix, AZ USA; ^2^ Abington Neurologic Associates, Abington, PA USA; ^3^ University of Washington Alzheimer's Disease Research Center, Seattle, WA USA; ^4^ Memory and Brain Wellness Center, University of Washington, Seattle, WA USA; ^5^ University of California San Diego, La Jolla, CA USA; ^6^ The Neuron Clinic, San Diego, CA USA; ^7^ Norton Neuroscience Institute, Louisville, KY USA; ^8^ Memory Treatment Centers, Jacksonville Beach, FL USA; ^9^ Memorial Healthcare Institute for Neuroscience, Owosso, MI USA; ^10^ New York University Langone Health, New York, NY USA; ^11^ Missouri Memory Center, Citizens Memorial Hospital, Bolivar, MO USA; ^12^ John P. and Kathrine G. McGovern Medical School at UTHealth, Houston, TX USA; ^13^ University of Miami Miller School of Medicine, Miami, FL USA; ^14^ Roaring Fork Neurology, Basalt, CO USA; ^15^ Eisai Inc, Nutley, NJ USA; ^16^ Eisai Inc., Nutley, NJ USA

## Abstract

**Background:**

Lecanemab‐irmb (LEQEMBI®) is indicated for the treatment of patients with Alzheimer’s disease (AD) in the mild cognitive impairment or mild dementia stage. Amyloid‐related imaging abnormalities (ARIA) are a known side effect of anti‐amyloid therapy and presence of the ε4 allele of apolipoprotein E (*APOE ε4*) increases the risk. ARIA is often transient and asymptomatic but can rarely lead to intracerebral hemorrhage (ICH; >1 cm); antithrombotic therapy can heighten the ICH risk, thus, caution is advised. This analysis describes lecanemab use in patients receiving antithrombotic therapy as well as in those homozygous for *APOE ε4*.

**Method:**

This multicenter, retrospective case series and patient pathway study was conducted in 15 geographically diverse neurology clinics, each abstracting deidentified medical chart data for up to 25 patients receiving lecanemab (≥7 infusions) and 1 neurologist per site completing an electronic survey plus an interview. Case report forms for patients who are homozygous for *APOE ε4* or receiving antithrombotic therapy were reviewed and descriptive statistics were run on these sub‐populations. This interim analysis (cutoff date: April 11, 2025) includes ∼25% of total expected cases (final cut: May 23, 2025). The protocol received central institutional review board exemption.

**Result:**

In this interim analysis of 94 cases, 18 patients were *APOE ε4* homozygous, and 18 were on antithrombotic therapy. All patients had at least 1 comorbidity at baseline. Among the *APOE ε4* homozygous group, 10 had baseline white matter hyperintensities, 1 case had microhemorrhages; ARIA‐E occurred in 3 patients. For those on anticoagulants, no abnormalities or ARIA were noted. In patients on antiplatelets, 11 had white matter intensities at baseline and 1 had microhemorrhages; ARIA occurred in 4 patients (1, ARIA‐H; 3, ARIA‐E). No cases of ICH were reported.

**Conclusion:**

In this interim analysis, ARIA‐E occurred in 17% of *APOE ε4* homozygous patients; no ARIA and no ICH was reported in those on concomitant anticoagulants. Given the rarity of ICH and small number of patients in this analysis being treated with anticoagulants, conclusions cannot be drawn. In the full data set analysis (data cutoff: May 23, 2025), ARIA risk by *APOE ε4* status and antithrombotic therapy use will be further explored.